# Black soldier fly larval oil as a renewable substrate for tailored PHA production

**DOI:** 10.1038/s41598-025-30033-1

**Published:** 2025-12-02

**Authors:** Shunmugham Keshaini, Idris Zainab-L, Kumar Sudesh

**Affiliations:** Ecobiomaterial Research Laboratory, School of Biological Sciences, Universiti Sains Malaysia, 11800 Penang, Gelugor Malaysia

**Keywords:** Black soldier fly larvae oil, Carbon source, *Cupriavidus necator*, Polyhydroxyalkanoates, Biochemistry, Biotechnology, Environmental sciences

## Abstract

Black soldier fly larvae (BSFL; *Hermetia illucens*) are environmentally friendly source of protein and also contain a significant amount of oil (BSFLO). BSFLO was compared with palm oil (PO) and crude palm kernel oil (CPKO) as new carbon sources for polyhydroxyalkanoate (PHA) synthesis using three *Cupriavidus necator* strains: H16 (wild type), Re2058/pCB113, and Re2160/pCB113 recombinants. BSFLO contained a relatively high amount of lauric acid (C12:0, 26.2%) and was suitable for cell growth and PHA synthesis. *C. necator* H16 produced more than 80 wt% poly(3-hydroxybutyrate) [P(3HB)] homopolymer with a cell dry weight (CDW) ranging from 8.52 to 9.26 g/L. To produce poly(3-hydroxybutyrate-*co*-3-hydroxyhexanoate) [P(3HB-*co*-3HHx)], Re2058/pCB113 showed a higher CDW (8.35–9.07 g/L) with 69.2–73.9 wt% P(3HB-*co*-3HHx), which exhibited Re2160/pCB113 with a CDW of 3.24–3.64 g/L and 65 wt% P(3HB-*co*-3HHx). The 3HHx composition of the PHA produced by all three strains utilizing BSFLO ranged from 0 to 28 mol%. The average molecular weight (*M*_w_) of the PHA was between 1.6 × 10^5^ and 15.1 × 10^5^ Da, and the melting temperature (*T*_m_) ranged from 81 °C to 171 °C. BSFLO is a potential feedstock for PHA synthesis and can be used in combination with other oils to regulate the 3HHx molar fraction.

## Introduction

The world demand for renewable and sustainable raw materials has intensified in response to escalating environmental pressures and international frameworks such as the United Nations Sustainable Development Goals (SDGs) highlighting SDG 12, which promotes responsible consumption and production, waste reduction, and the transition toward renewable resource utilization^1^. Particularly, the global transition towards natural alternatives in plastics manufacturing has generated interest in creating biodegradable polymers.

Though petrochemical-derived plastic materials are vital in everyday life, they are dependent on non-renewable resources and result in waste accumulation which contribute to pollution and waste management challenges. Plant-derived renewable materials such as polylactic acid (PLA), polybutylene succinate (PBS), bio-polyethylene (bio-PE), and polyhydroxyalkanoates (PHAs) are some of the eco-friendly options under development^2^. While PLA and PBS can be brittle and costly, and bio-PE is non-biodegradable, PHAs are fully biosynthetic and biodegradable in various natural environments, including oceans. Among these, PHAs have gained increasing attention because they are fully biosynthetic, biodegradable, and hold potential as a sustainable alternative to non-biodegradable, petrochemical-derived synthetic plastics.

PHAs are biocompatible and biodegradable polyesters that are produced naturally by many microorganisms under nutrient deficiency (especially of nitrogen, phosphorus, magnesium, sulphur, or oxygen) and an abundance of carbon sources^3,4^. The intracellular carbon and energy reserve materials are produced when microbes store available carbon sources during limiting nutrient conditions^5^. Among microbial producers, *Cupriavidus necator* is extensively studied owing to its superior productivity and robustness in PHA biosynthesis^6^. The choice of carbon sources is crucial for PHA production because it directly affects yield, cost, and sustainability. Common carbon substrates include industrial waste, such as glycerol, molasses, and waste cooking oils, as well as monosaccharides, fatty acids, and plant oils^7,8^. Lipid substrates, such as palm (0.72 to 1 g PHA per g/L)^9,10^ and soybean oil (0.72 to 0.76 g PHA per g/L)^6^, are also frequently used due to their liquid state and suitable fatty acid composition. These characteristics are important because they determine the monomer composition during PHA copolymer synthesis^8,11^. Fatty acids like lauric acid, for instance, can potentially stimulate the synthesis of monomers like 3-hydroxyhexanoate (3HHx), providing PHA copolymers with improved flexibility and thermal robustness. This ultimately yields more resilient, elastic, and versatile bioplastics.

However, the widely studied lipid substrates are edible and precious commercial oils primarily used by food processing industries^6,12,13^. Their large-scale use for biopolymer production is unsustainable, as it reduces cooking oil availability, raises prices, and intensifies food–feed competition^14^.

Despite these advancements, there are still challenges in reducing the reliance on edible carbon sources to produce PHA. Thus, over the years, various plant oils and waste-derived oils have been explored as potential substrates to support the microbial synthesis of PHAs. However, many of these sources still compete, directly or indirectly, with food and feed industries. Despite the increasing interest in alternative lipid sources, the potential of black soldier fly larval oil (BSFLO; *Hermetia illucens*) for PHA biosynthesis remains underexplored. Black soldier fly larvae are well known for their ability to convert organic waste into nutrient-rich biomass, and they play a significant ecological role in waste bioconversion^15^. *H. illucens*, a true fly of the order Diptera (Stratiomyidae), has attracted attention for its global adaptability and potential for sustainable waste valorization^16,17^. Its larvae effectively convert a range of organic wastes into valorized biomass of high value, thereby aiding greenhouse gas mitigation and circular bioeconomy initiatives. Moreover, BSFL biomass contains high protein and lipid contents, making it a multi-purpose source^18,19^.

BSFLO is either harvested as a main product from insect farm operations aimed at oil processing or as a by-product when producing protein meal, where oil is extracted from the residual larval biomass^15,20,21^. BSFLO contains 28–40% lipid, rich in lauric and palmitic acids^18^, valuable for microbial metabolism, particularly in regulating 3HHx monomer formation^22,23^. In addition to *C. necator*, BSFLO provides a valuable carbon source for various PHA-producing microorganisms that prefer lipid substrates. Beyond *C. necator*, other PHA-producing strains such as *Pseudomonas putida*^24^ and *Alcaligenes latus* have demonstrated efficient fatty acid utilization through their enzymatic activities suggests the potential versatility of BSFLO as a raw material in biotechnological processes aimed at substituting petrochemical plastics with environmentally friendly alternatives.

This study aimed to extract and characterize black soldier fly larval oil (BSFLO) and to evaluate its suitability as a carbon source for PHA production by *C. necator* H16 and its recombinant strains Re2058/pCB113 and Re2160/pCB113, with the expectation that BSFLO could support PHA copolymer synthesis comparable to that achieved using conventional plant oils.

## Material and methods

### Bacterial strain and media

The bacterial strains used in this study were *C. necator* wild-type H16 and the recombinant strains Re2058/pCB113 and Re2160/pCB113, which carry plasmid pCB113 and are capable of producing P(3HB-*co*-3HHx)^25^. For routine maintenance and inoculum preparation, these bacteria were cultivated in nutrient-rich (NR) medium containing 10 g/L of meat extract, 10 g/L of peptone and 2 g/L of yeast extract^26^. For PHA biosynthesis, mineral salts medium (MM) was used, and its composition is detailed in the biosynthesis section.

### Insect collection, processing and storing

The live BSFL were procured from a commercial source in Penang, Malaysia. The larvae were washed with distilled water to remove food residue and faecal pellets, dried with paper towels. They were subsequently euthanized by freezing at –20℃ for further analysis to preserve tissue integrity for oil extraction.

### BSFL drying methods

The frozen larvae were thawed and the initial moisture content with the weight of beakers was weighed in advance. 100 g of thawed larvae were subjected to freeze-drying using a Labconco Free Zone freeze dryer (USA) for 24–36 h until a constant weight was achieved, and the moisture content was measured. The freeze-dried samples were ground using a pestle and mortar and then sieved through a 1 mm screen sieve to obtain a homogenized, fine, and uniform powder.

### BSFL oil extraction

The conventional approach for the oil extraction process was carried out with minor modifications^27–29^. The BSFL powder was dissolved in hexane at a ratio of 1:15 (w/v), and the extraction was done in a fume hood for 72 to 96 h to ensure complete lipid recovery from the freeze-dried larvae. The resulting oil-hexane mixture was first filtered using a No. 1 Whatman filter paper with the aid of a vacuum pump. The filtrate was then concentrated using a rotary evaporator and left in the fume hood until a constant weight was achieved. The oil yield was calculated using Eq. 1^30^:

1 $$\text{Oil yield }(\text{\%}) = \frac{\text{Mass of extracted oil }}{\text{Mass of dried material}} \times 100\text{\%}$$

### Carbon sources

Three different oils (Table 1) were used in this study. The palm olein (PO) was from the Vesawit cooking oil brand which is a high-quality palm olein. The crude palm kernel oil (CPKO) was from IOI Acidchem Pte. Ltd. BSFLO was extracted using maceration with hexane extraction method described earlier. PO, CPKO, and BSFLO were autoclaved separately and aseptically added to the culture medium at the desired concentrations.

**Table 1:** The fatty acid composition of BSFLO, mealworm oil and palm oils.

Fatty Acids	BSFL Oil^a^	Mealworm Oil	PO^a^*	CPKO^a^*	BSFL oil (literature) **
Lauric acid (C12:0)	26.2	0.2	0.2	48.3	23.4- 48.4
Myristic acid (C14:0)	7.3	3.1	1.0	15.5	3.3- 9.9
Palmitic acid (C16:0)	20.5	16.1	35.8	8.0	10.5- 21.9
Palmitoleic acid (C16:1)	2.3	0.1	0.1	-	2.3- 7.6
Stearic acid (C18:0)	4.6	3.9	4.1	2.1	1.8- 5.3
Oleic acid (C18:1n9)	27.6	55.9	43.8	15.4	10.2- 27.1
Linoleic acid (C18:2n6)	9.8	20.7	14.3	2.6	3.22- 13.0
(C18:3n3)	0.5	-	0.2	-	0.1–1.7
Arachidic acid (C20:0)	0.7	-	0.4	-	0.04–0.6
Behenic acid (C22:0)	0.6	-	-	-	-
ƩSFA^a^	59.9	23.3	41.5	73.9	
ƩMUFA^a^	29.9	56.0	43.9	15.4	
ƩPUFA^a^	10.3	20.7	14.5	2.6	

^a^SFA, saturated fatty acid; MUFA, monounsaturated fatty acid; PUFA, polyunsaturated fatty acid; BSFL, black soldier fly larvae; PO, palm olein; CPKO, crude palm kernel oil.

*Ref.^13,31^

**Ref.^32,33^

### Fatty acid methyl ester analysis

The fatty acid methyl ester (FAME) was prepared according to AOAC (1990)^34^ standards. Approximately 100 mg of the extracted BSFLO was placed in a 50 mL flask. Then, 4 mL of 2% (v/v) methanolic sodium hydroxide was added, and the mixture was refluxed at 70 °C for 3 min. Subsequently, 5 mL of 1.3 M boron trifluoride (BF₃)/methanol reagent was added, and heating continued for 3 min^35,36^. About 4 mL of 99% n-heptane was then added to the boiling mixture and heated for 1 min.

The flask was removed from the water bath and allowed to cool to room temperature. A saturated sodium chloride solution (35 g NaCl in 100 mL distilled water) was added up to 50 mL for layer separation. The upper heptane layer (~ 1 mL), containing the fatty acid methyl esters, was transferred into a screw-cap vial using a Pasteur pipette, wrapped in aluminum foil and stored at − 20 °C.

FAME analysis was conducted using a Shimadzu GC-2010 equipped with a flame ionization detector. Esters were separated on an SPTM 2560 fused silica capillary column (100 m length, 0.25 mm internal diameter, 0.2 µm film thickness; Supelco, Bellefonte, PA, USA). Nitrogen was used as the carrier gas (3 mL min⁻^1^), and samples were injected with a SPL-2010 injector (split ratio 1:100). The injector and detector temperatures were set at 250 °C. The column temperature was ramped from 140 °C to 240 °C at 2 °C min⁻^1^. Fatty acid compositions were identified by comparing retention times with a standard (Supelco 37 Component FAME mix, PUFA No. 2).

### P(3HB) and P(3HB-co-3HHX) synthesis in shake flasks with PO + BSFLO and CPKO + BSFLO as carbon sources

The PHA biosynthesis was carried out in one-stage batch fermentation in shake flasks. Two loops of bacterial cells were inoculated into NR medium and cultivated at 30 °C, 200 rpm in an incubator shaker. Upon reaching an OD₆₀₀ of 4.5–5.0, 1.5 mL (3%) of culture was transferred into 50 mL of mineral medium (MM) in a 250 mL Erlenmeyer flask.

The MM composition was as follows: 4.6 g/L NaH₂PO₄, 4.0 g/L Na₂HPO₄, 0.45 g/L K₂SO₄, 0.39 g/L MgSO₄, and 62 mg/L CaCl₂. MgSO₄ and CaCl₂ were autoclaved separately to prevent precipitation and added to the MM before inoculation. The trace elements, 15.0 g/L FeSO₄·7H₂O, 2.4 g/L MnSO₄·H₂O, 0.48 g/L CuSO₄·5H₂O, 2.4 g/L ZnSO₄·7H₂O, were dissolved in 0.1 M HCl^25^, filter-sterilized, and incorporated into the MM prior to inoculation.

Urea (0.54 g/L) was used as the nitrogen source. Various oil mixtures (PO + BSFLO and CPKO + BSFLO) were supplied at 10 g/L to the three different strains to assess PHA production. Oils and urea were autoclaved separately and added to the MM before inoculation.

The inoculated flasks were incubated at 30 °C, 200 rpm for 48 h^25,26^. After cultivation, cells were harvested by centrifugation at 10,000 × g, 4 °C for 10 min (KUBOTA 2100, Japan). Cell pellets were washed with 20 mL n-hexane and 20 mL distilled water, centrifuged under the same conditions, and then washed twice with distilled water. The washed cells were resuspended in ~ 1 mL distilled water. Resuspended cells were stored at − 20 °C and freeze-dried at − 40 °C for 72 h. The resulting cell dry weight was measured.

### Gas chromatography (GC) analysis

The amount and monomer compositions of PHA in the cells were quantified using gas chromatography (GC). Approximately, 12–20 mg of the freeze-dried cells were subjected to methanolysis in the presence of 15% (v/v) of sulphuric acid and 85% (v/v) of methanol for 140 min at 100 °C^37^.

The mixture was cooled to room temperature then 1 mL distilled water was added and vortexed for 1 min. The mixture was allowed to separate into two layers. The bottom chloroform layer was carefully aspirated by using a Pasteur pipette and added to a small amount of sodium sulfate anhydrous in a flat-bottom glass vial to absorb traces of water.

A 0.5 mL of the solution was pipetted into a screw-cap GC vial and added with a 0.5 mL caprylate methyl ester (CME) solution as an internal standard. The sample was then analyzed using a GC-2010 AF 230 LV (Shimadzu, Japan) equipped with a capillary column SPB-1 (30 m length, 0.25 mm internal diameter and 0.25 µm film thickness; Supelco, Bellefonte, PA, USA) connected to a flame ionization detector. The carrier gas was nitrogen gas (1 mL min−^1^), and 2 µL of sample in chloroform was injected via auto-injector (Shimadzu AOC-20i). The injector and detector temperature were 270 °C and 280 °C respectively. The column temperature rose from 70 °C to 280 °C at 10 °C min−^1^.

### Polymer extraction and purification

The freeze-dried bacterial cells were refluxed in chloroform for 4 h at 60 °C. The ratio of the weight of freeze-dried cells to the volume of chloroform was fixed at 1:150^38,39^. The refluxed solution was cooled to room temperature before the cell debris was filtered out using a No. 1 Whatman filter paper. Then, the filtrate was concentrated to 10 mL using a rotary evaporator which was then precipitated and purified by dripping the concentrated solution into 100 mL of rapidly stirred cold methanol. The precipitated polymer was filtered through a PTFE membrane filter to separate it from methanol. The extracted polymers were dried in 50 °C oven overnight.

### Gel permeation chromatography (GPC) analysis

The molecular weights of PHA were measured using Gel Permeation Chromatography (GPC). Purified PHA samples (2–3 mg) were dissolved at a concentration of 1 mg/mL chloroform and filtered using a 0.45 μm PVDF membrane^40^. The GPC analysis was conducted using a GPC machine at a column oven temperature of 40 °C with chloroform as the eluent at a flow rate of 0.8 mL/min^41^. The weight-average molecular weight (*M*_w_), number-average molecular weight (*M*_n_), and polydispersity index (*M*_w_/*M*_n_) were determined from the resulting chromatogram.

### Differential scanning calorimetry (DSC)

The thermal analysis of the polymers was performed using a Perkin Elmer Pyris 1 Differential Scanning Calorimetry (DSC) system following the method described^22^. Approximately 3 mg of each sample was sealed hermetically in an aluminum DSC pan and scanned from −30 °C to 200 °C at a constant heating rate of 20 °C/min. The analysis was carried out in a nitrogen atmosphere. The DSC thermogram of the second scan was used to determine the glass transition temperature (*T*_g_) and crystalline melting point (*T*_m_).

### Phase contrast observation

The bacterial cells were observed under an Olympus BX41 microscope (Olympus, Japan), phase contrast microscope. Clean glass slides were prepared with a small drop of bacterial suspension on them and covered with a cover slip. The prepared glass slide was subsequently placed on the microscope stage, and the cells were observed under the lowest magnification up to the highest magnification lens, 100 × with oil immersion.

### Statistical analysis

The experimental data in this study are presented as mean ± standard deviation (n = 3). The data were assessed using one-way analysis of variance (ANOVA) and Tukey’s Honestly Significant Differences (HSD) test, also known as Tukey’s test, to determine significant differences among the concentrations of carbon sources.

Differences among the means of each concentration for the same measurements, such as cell dry weight (CDW) and PHA content, were compared. Differences with P > 0.05 were considered statistically not significant. All statistical analyses were performed using GraphPad Prism software, version 10.1.2 for Windows.

## Results & discussion

### The total lipid content and fatty acid compositions of BSFLO

The total lipid content of freeze-dried black soldier fly larvae (BSFL) was approximately 29%, determined using the *n*-hexane maceration method. This value is similar to the 28.4% lipid content reported during the larval stage in previous studies^42^. This value lies within the range of the 18% to 48% lipid content reported in previous studies^17,20,43,44^. The lipid content of freeze-dried mealworm larvae was approximately 23%, which is lower than BSFL due to differences in fat storage efficiency. Typically, BSFL have higher lipid content than mealworms due to efficient fat storage when grown on organic waste. During the prepupal stage, BSFL stock energy in the form of fats to support growth, mating, and reproduction^45^.

Variations in lipid content among studies^17,20,43^ are influenced not only by extraction methods but also by the types of lipids recovered. Previous research^46^ showed that fatty acid profiles of seven insect species were minimally affected by the polarity of extraction solvents (e.g., hexane, ethanol, acetone).

As reported previously^47^, variations in lipid content and fatty acid profiles of BSFL are strongly influenced by the nutritional composition of the larval diet, dietary fat levels, substrate type, and rearing conditions applied during growth^48,49^.

The fatty acid profile of BSFLO, along with mealworm oil, PO, and CPKO, is presented in Table 1. The fatty acids with a carbon chain length of 12–18 carbons (C12:0 to C18:3) were found in both insect oils. The fatty acid profile observed in this work aligns in part with the reported findings^49^, especially in terms of the key fatty acids present. In the current analysis, the oleic acid content was the highest, at 27.6%, followed by lauric acid and palmitic acid at 26.2% and 20.5%, respectively, with the lowest levels in linoleic (C18:3n3) accounting for just 0.5% of the total fatty acids. By comparison^50^, reported lauric acid as the most dominant component at 52%, with palmitic acid ranging from 12 to 22% and oleic acid between 10 and 25%. While lauric, palmitic, and oleic acids were predominant in both studies, their proportions differed, likely due to feed formulation, rearing conditions, or larval maturity^48,51,52^. These results also aligned with the previous findings^53^ as lauric acid is one of the major and common saturated fatty acid present in BSFLO.

In contrast, mealworm oil had the highest levels of oleic acid (C18:1n9, 55.9%) and linoleic acid (C18:2n6, 20.7%), with the lowest levels of palmitoleic acid (C16:1presentss, consistent with published data^54^.

Mealworm oil has a similar composition to various vegetable oils, such as rice bran and peanut oils, with oleic, linoleic, and palmitic acids as the main fatty acids. Similarly, both house crickets (*Acheta domesticus*) and grasshoppers (*Chortippus parallels*) have fatty acid profiles dominated by oleic and linoleic acids, with grasshoppers additionally containing α-linolenic acid. Typical insect oils are dominated by unsaturated fatty acids, but BSFLO has far more saturated fatty acids, particularly lauric acid^46^. Based on Table 1, BSFLO contains 60% saturated fatty acids, higher than mealworm oil (23%) and PO (42%), but similar to CPKO (74%). The elevated saturated fatty acid content in BSFLO is likely due to the non-feeding adult stage, where energy is stored as oxidation-resistant fatty acids^55^.

### Biosynthesis of P(3HB) homopolymer and P(3HB-co-3HHx) copolymer by C. necator H16, Re2058/pCB113 and Re2160/pCB113 with PO and BSFLO as carbon sources

The biosynthesis of P(3HB) and P(3HB-*co*-3HHx) was explored using *C. necator* strains (H16, Re2058/pCB113, and Re2160/pCB113), with PO and BSFLO as carbon sources. The influence of varying PO and BSFLO concentrations on PHA yield, and composition was examined in all three strains.

#### Biosynthesis of P(3HB) homopolymer by *C. necator* H16 with PO and BSFLO as carbon sources

The ability of *C. necator* H16 to produce P(3HB) was examined using PO, BSFLO, and their various mixtures as the carbon sources, each provided at a concentration of 10 g/L. As shown in Table 2, the wild type strain successfully grew and accumulated PHA under all conditions, though some minor variations in biomass and polymer content were observed depending on the carbon source used. All carbon sources demonstrated excellent 1:1 oil conversion efficiency, with PO-based and blended treatments giving the best results. Among the tested substrates, the cultures grown with 100% PO achieved the highest dry cell weight (DCW) of 10.74 ± 0.09 g/L, with a PHA content of 84.8 ± 0 wt.%. Mixtures of PO and BSFLO also supported comparable levels of PHA accumulation. For example, a 50:50 mixture resulted in a DCW of 10.16 ± 0.64 g/L and a PHA content of 80.3 ± 0.01 wt.%. In contrast, when BSFLO was used as the sole carbon source, the biomass yield was lower, at 8.52 ± 0.64 g/L, but the PHA content remained high at 82.2 ± 0.01 wt.%.

**Table 2:** Polyhydroxyalkanoate production in *C. necator* H16 by using different carbon sources^a^.

Carbon Sources (10 g/L)	Dry Cell Weight (g/L)	PHA Content^b^ (wt.%)	PHA Composition^b^ (mol%)	
			3HB	3HHx
PO 100%	10.74 ± 0.09^x^	84.8 ± 0^x^	100 ± 0	0 ± 0
PO 75% + BSFLO 25%	10.09 ± 0.03^x^	79.1 ± 0.01^x^	100 ± 0	0 ± 0
PO 50% + BSFLO 50%	10.16 ± 0.64^x^	80.3 ± 0.01^x^	100 ± 0	0 ± 0
PO 25% + BSFLO 75%	10.01 ± 0.43^x^	79.0 ± 0^x^	100 ± 0	0 ± 0
BSFLO 100%	8.52 ± 0.64^y^	82.2 ± 0.01^x^	100 ± 0	0 ± 0

^a^The cells were cultivated in 50 mL MM supplemented with 10 g/L of oil as carbon source and 0.54 g/L of urea as nitrogen source at 30 °C, 200 rpm for 48 h. The values reported are means ± standard deviations from triplicate cultures. The values indicated by different superscript alphabets (x, y) are significantly different (Tukey’s HSD test, p˂0.05).

^b^Determined by Gas chromatography. PHA, polyhydroxyalkanoate; 3HB, 3-hydroxybutyrate; 3HHx, 3-hydroxyhexanoate; PO, Palm olein; BSFLO, Black soldier fly larval oil.

Across all the carbon sources, the PHA produced was composed entirely of 3-hydroxybutyrate (3HB), with no detection of 3-hydroxyhexanoate (3HHx) monomers. This outcome confirms that the wild-type strain preferentially synthesizes short-chain-length PHA. *C. necator* H16 naturally synthesizes short-chain-length PHA, governed by the native phaCAB operon. This operon directs metabolic flux toward P(3HB) homopolymer.

**Fig. 1:**
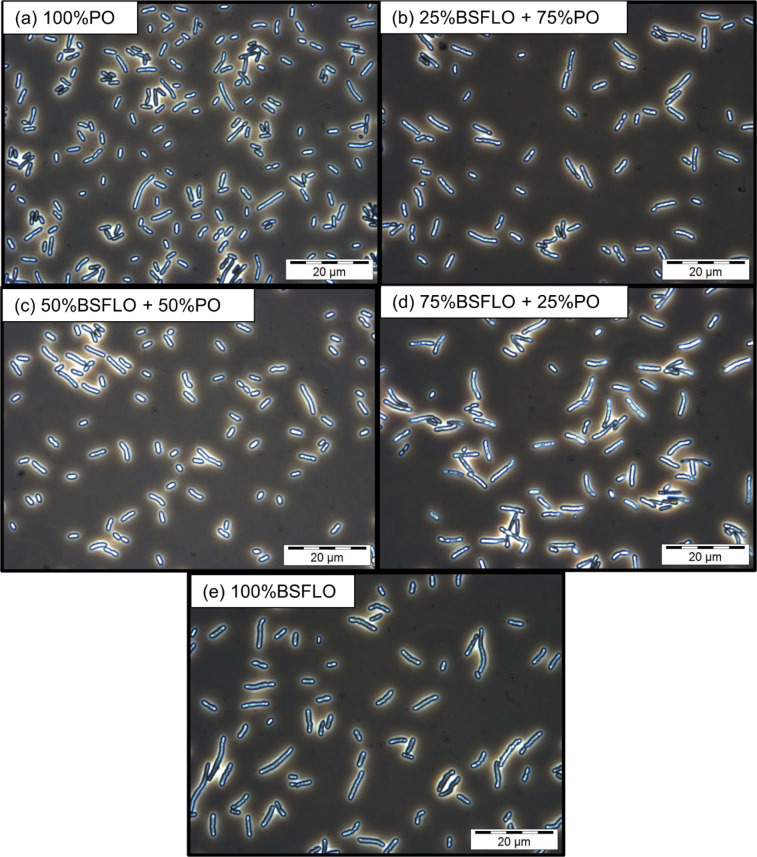
Phase contrast microscope images at 1000 × magnification of P(3HB) in *C. necator* H16 using (**a**) 100% PO, (**b**) 25% BSFLO + 75% PO, (**c**) 50% of BSFLO + 50% PO, (**d**) 75% of BSFLO + 25% PO and (**e**) 100% of BSFLO.

Since this is the first time BSFLO was used as a carbon source, the cell morphology and intracellular PHA accumulation was examined via phase contrast microscopy at 1000 × magnification (Fig. 1**)**. Cells cultivated with 100% PO (Fig. 1a) appeared densely populated and contained large, bright inclusion bodies, typical of PHA granules. Similar granule formation was observed in PO-BSFLO mixtures (Fig. 1b–d), though subtle differences in granule size and density were apparent. Cells grown with 100% BSFLO (Fig. 1e) also contained prominent PHA granules, despite slightly lower cell density, consistent with the lower biomass yield.

Statistical evaluation using Tukey’s HSD test confirmed that the reduction in biomass observed with 100% BSFLO was statistically significant compared to the PO-based treatments (P < 0.05). No significant differences in PHA content were observed among the substrates (P > 0.05), indicating that while BSFLO may influence growth, it does not compromise PHA accumulation. These results demonstrate that *C. necator* H16 can reliably produce P(3HB) using PO, BSFLO, or their combinations, highlighting BSFLO’s potential as an efficient feedstock for PHA production.

#### Biosynthesis of P(3HB-*co*-3HHx) copolymer by *C. necator* Re2058/pCB113 with PO and BSFLO as carbon sources

*C. necator* Re2058/pCB113 produced P(3HB-*co*-3HHx) when grown on PO, BSFLO, or their mixtures (10 g/L). Table 3 shows that all substrates supported biomass growth and PHA accumulation. However, notable differences were observed among treatments. DCW ranged from 9.07 ± 0.01 g/L (100% BSFLO) to 10.39 ± 0.50 g/L (75% PO + 25% BSFLO). This indicates that BSFLO alone produces less biomass than PO-based mixtures. However, the PHA content was the highest in cultures with 25% PO + 75% BSFLO (84.3 ± 1.67 wt.%) and 50% PO + 50% BSFLO (80.6 ± 0.27 wt.%). These values were significantly higher than 100% BSFLO (69.2 ± 1.73 wt.%), which showed the lowest PHA accumulation (P < 0.05).

**Table 3:** Polyhydroxyalkanoate production in *C. necator* Re2058/pCB113 using different carbon sources^a^ .

Carbon Sources (10 g/L)	Dry Cell Weight (g/L)	PHA Content^b^ (wt.%)	PHA Composition^b^ (mol%)	
			3HB	3HHx
PO 100%	10.33 ± 0.34^x^	79.0 ± 1.0^x^	86 ± 1	14 ± 1
PO 75% + BSFLO 25%	10.39 ± 0.50^x^	74.7 ± 2.4^xy^	85 ± 2	15 ± 2
PO 50% + BSFLO 50%	9.26 ± 0.42^xy^	80.6 ± 0.3^x^	85 ± 0	15 ± 0
PO 25% + BSFLO 75%	9.61 ± 0.70^xy^	84.3 ± 1.7^x^	84 ± 2	16 ± 2
BSFLO 100%	9.07 ± 0.01^y^	69.2 ± 1.7^y^	82 ± 2	18 ± 2

^a^The cells were cultivated in 50 mL MM supplemented with 10 g/L of oil as carbon source and 0.54 g/L of urea as nitrogen source at 30 °C, 200 rpm for 48 h. The values reported are means ± standard deviations from triplicate cultures. The values indicated by different superscript alphabets (x, y) are significantly different (Tukey’s HSD test, p˂0.05).

^b^Determined by Gas chromatography.

PHA, polyhydroxyalkanoate; 3HB, 3-hydroxybutyrate; 3HHx, 3-hydroxyhexanoate; PO, Palm olein; BSFLO, Black soldier fly larval oil.

Adding 25% BSFLO increased 3HHx from 14 mol% (100% PO) to 15 mol% in the mixture, reaching 18 mol% with 100% BSFLO. This pattern suggests that the medium-chain fatty acids in BSFLO, particularly lauric acid, enhance the supply of precursors for 3HHx synthesis, supporting previous observations^25^ that fatty acid-rich substrates improve 3HHx formation.

**Fig. 2:**
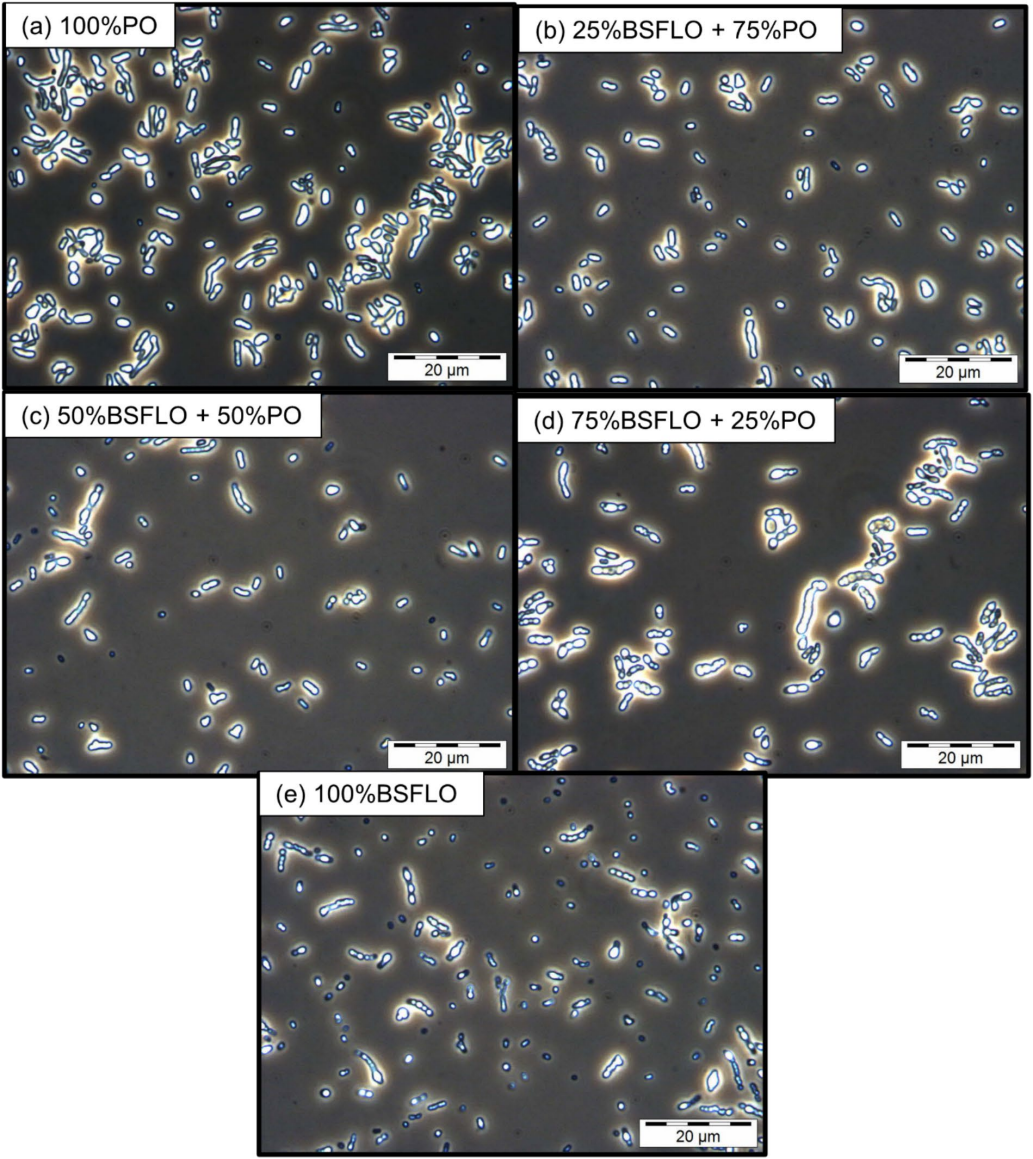
Phase contrast microscope images at 1000 × magnification of P(3HB-*co*-3HHx) in Re2058/pCB113 using (**a**) 100% PO, (**b**) 25% BSFLO + 75% PO, (**c**) 50% of BSFLO + 50% PO, (**d**) 75% of BSFLO + 25% PO and (**e**) 100% of BSFLO.

Phase contrast microscopy at 1000 × magnification (Fig. 2) provided visual confirmation of intracellular PHA accumulation, with bright granules observed in all samples. Cells from 75% BSFLO and 25% PO (Fig. 2d) were dense and clumped together, similar to those in 100% PO (Fig. 2a). In contrast, cells with BSFLO content (Fig. 2b, c, and e) were more dispersed but still showed clear granule formation. Even under 100% BSFLO conditions (Fig. 2e), PHA inclusions were evident, though at relatively lower polymer content, indicating BSFLO’s ability to support copolymer formation.

#### Biosynthesis of P(3HB-*co*-3HHx) copolymer by *C. necator* Re2160/pCB113 with PO and BSFLO as carbon sources

*C. necator* Re2160/pCB113 produced P(3HB-*co*-3HHx) when grown on PO, BSFLO, or mixtures (10 g/L), with all substrates supporting PHA accumulation, though DCW and PHA content varied significantly depending on the substrate (Table 4). The maximum biomass yield was achieved in 100% PO (5.95 ± 0.31 g/L), and the minimum in 100% BSFLO (3.64 ± 0.32 g/L). Statistical analysis revealed a significant decline in cell growth with increasing BSFLO concentration. Similarly, the PHA content was also affected, ranging from 66.4 ± 1.03 wt.% for the PO-only treatment to 58.9 ± 0.64 wt.% for the 25% BSFLO, before increasing again to 71.0 ± 1.00 wt.% for the 75% BSFLO mixture.

**Table 4:** Polyhydroxyalkanoate production in *C. necator* Re2160/pCB113 using different carbon sources^a^.

Carbon Sources (10 g/L)	Dry Cell Weight (g/L)	PHA Content^b^ (wt.%)	PHA Composition^b^ (mol%)	
			3HB	3HHx
PO 100%	5.95 ± 0.31^x^	66.4 ± 1.0^x^	78 ± 1	22 ± 1
PO 75% + BSFLO 25%	4.58 ± 0.81^xy^	58.9 ± 0.6^x^	76 ± 1	24 ± 1
PO 50% + BSFLO 50%	4.30 ± 0.62^y^	61.7 ± 0.3^x^	74 ± 0	26 ± 0
PO 25% + BSFLO 75%	3.97 ± 0.57^y^	71.0 ± 1.0^x^	75 ± 1	25 ± 1
BSFLO 100%	3.64 ± 0.32^y^	65.5 ± 0.2^x^	73 ± 0	27 ± 0

^a^The cells were cultivated in 50 mL MM supplemented with 10 g/L of oil as carbon source and 0.54 g/L of urea as nitrogen source at 30 °C, 200 rpm for 48 h. The values reported are means ± standard deviations from triplicate cultures. The values indicated by different superscript alphabets (x, y) are significantly different (Tukey’s HSD test, p˂0.05).

^b^Determined by Gas chromatography.

PHA, polyhydroxyalkanoate; 3HB, 3-hydroxybutyrate; 3HHx, 3-hydroxyhexanoate; PO, Palm olein; BSFLO, Black soldier fly larval oil.

Although BSFLO caused a reduction in biomass, it positively influenced 3HHx incorporation. The 3HHx monomer fraction increased progressively from 22 mol% in 100% PO to 27 mol% in 100% BSFLO. Even a small infusion of BSFLO into PO (as low as 25%) led to a measurable increase in 3HHx content, indicating a shift in metabolic activity toward copolymer formation, which is consistent with trends observed in the Re2058/pCB113 strain.

**Fig. 3:**
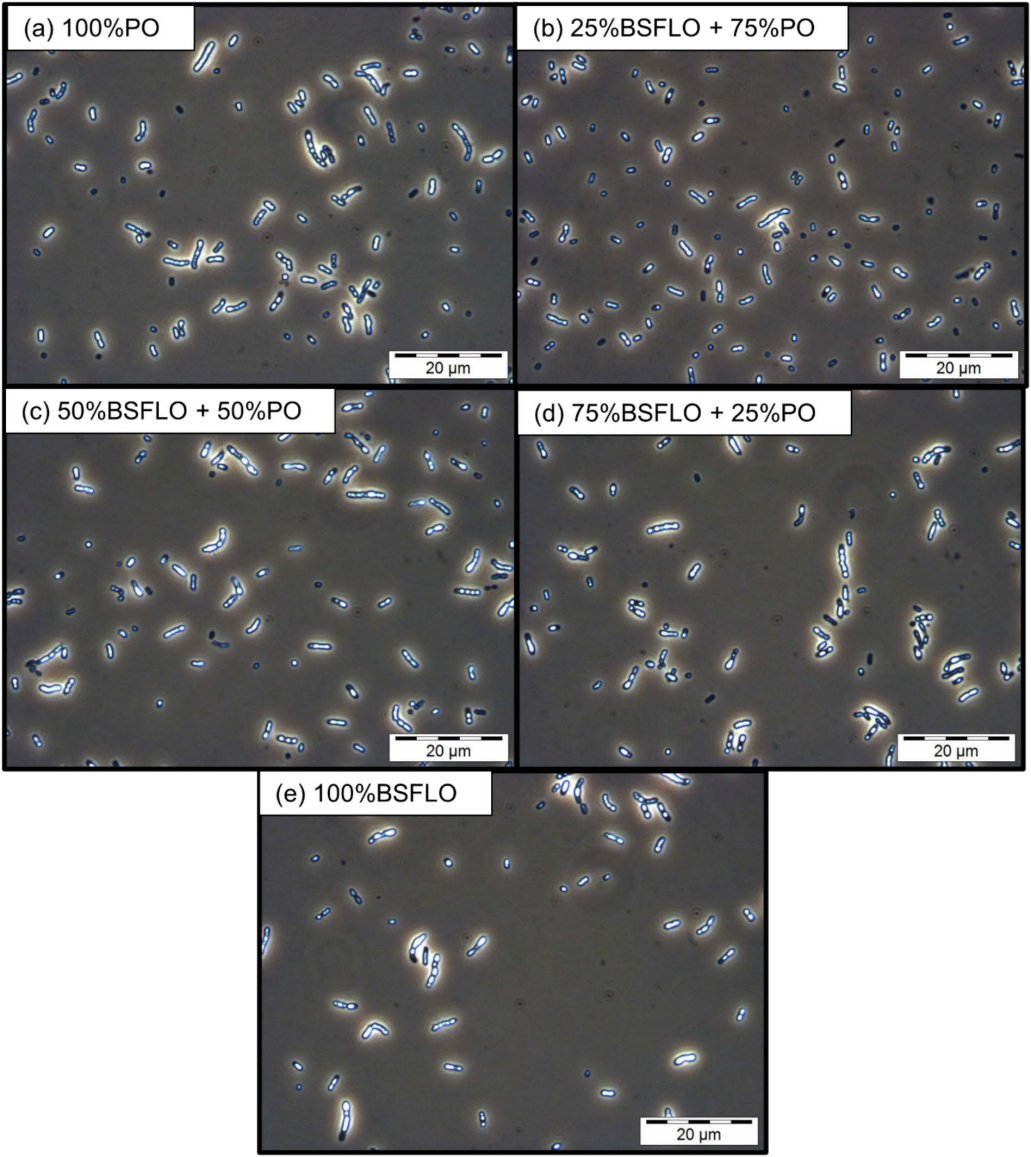
Phase contrast microscope images at 1000 × magnification of P(3HB-*co*-3HHx) in Re2160/pCB113 using (**a**) 100% PO, (**b**) 25% BSFLO + 75% PO, (**c**) 50% of BSFLO + 50% PO, (**d**) 75% of BSFLO + 25% PO and (**e**) 100% of BSFLO.

The phase contrast microscopy images (Fig. 3) show that PO-grown cells (Fig. 3a) were more clumped, while BSFLO-treated cells (Fig. 3b–e) were more dispersed with smaller clusters, yet all samples exhibited clearly visible PHA granules. The similar brightness and size of the inclusion bodies between treatments suggest that, despite differences in cell density, PHA accumulation was comparable, supporting the biochemical results that showed no significant differences in PHA content between substrates.

### Biosynthesis of P(3HB) homopolymer and P(3HB-co-3HHx) copolymer by C. necator H16, Re2058/pCB113 and Re2160/pCB113 using CPKO and BSFLO as carbon sources

The biosynthesis of P(3HB) and P(3HB-*co*-3HHx) was investigated in *C. necator* strains (H16, Re2058/pCB113, and Re2160/pCB113), with CPKO and BSFLO as carbon sources. The influence of varying CPKO and BSFLO concentrations on polymer yield and composition was examined to evaluate PHA production.

#### Biosynthesis of P(3HB) homopolymer by *C. necator* H16 using CPKO and BSFLO as carbon sources

The biosynthesis of P(3HB) by *C. necator* H16 was studied using CPKO, BSFLO, and their mixtures as sole carbon sources at 10 g/L. As shown in Table 5, the strain efficiently accumulated PHA under all tested conditions. The resulting polymer consisted entirely of 3HB, confirming that *C. necator* H16 synthesized a P(3HB) homopolymer. The DCW ranged from 9.26 ± 0.89 g/L under 100% BSFLO to 10.59 ± 0.40 g/L under 50:50 CPKO + BSFLO. The lowest biomass was observed with 100% BSFLO, but statistical analysis revealed that there were no significant differences in both DCW and PHA content (P > 0.05) between all carbon sources. PHA content was equally maintained across all treatments, ranging from 81.2 ± 0.01 wt.% in BSFLO to 86.2 ± 0 wt.% in the CPKO + BSFLO (50:50) blend.

**Table 5:** Polyhydroxyalkanoate production in *C. necator* H16 by using different carbon sources^a^ .

Carbon Sources (10 g/L)	Dry Cell Weight (g/L)	PHA Content^b^ (wt.%)	PHA Composition^b^ (mol%)	
			3HB	3HHx
CPKO 100%	10.39 ± 0.14^x^	84.9 ± 0.02^x^	100 ± 0	0 ± 0
CPKO 75% + BSFLO 25%	10.30 ± 0.42^x^	84.3 ± 0.01^x^	100 ± 0	0 ± 0
CPKO 50% + BSFLO 50%	10.59 ± 0.40^x^	86.2 ± 0^x^	100 ± 0	0 ± 0
CPKO 25% + BSFLO 75%	10.41 ± 0.29^x^	85.3 ± 0^x^	100 ± 0	0 ± 0
BSFLO 100%	9.26 ± 0.89^x^	81.2 ± 0.01^x^	100 ± 0	0 ± 0

^a^The cells were cultivated in 50 mL MM supplemented with 10 g/L of oil as carbon source and 0.54 g/L of urea as nitrogen source at 30 °C, 200 rpm for 48 h. The values reported are means ± standard deviations from triplicate cultures. All the values indicated superscript alphabet (x) are not significantly different (Tukey’s HSD test, p˂0.05).

^b^Determined by Gas chromatography.

PHA, polyhydroxyalkanoate; 3HB, 3-hydroxybutyrate; 3HHx, 3-hydroxyhexanoate; CPKO, Crude palm kernel oil; BSFLO, Black soldier fly larval oil.

In general, *C. necator* H16 displayed consistent and high DCW and PHA content across both CPKO- and PO-based substrates. When cultivated with 50% CPKO and BSFLO, H16 achieved the highest biomass (10.59 g/L) and PHA content (86.2 wt.%), and similarly high values were observed with 100% PO (10.74 g/L and 84.8%) and 75% BSFLO mixture with 25% of CPKO (10.41 g/L and 85.3 wt.%). The addition of BSFLO in varying ratios to either CPKO or PO did not significantly alter the PHA production, with yields ranging from 79.0 wt.% to 86.2 wt.%.

The findings indicate that *C. necator* H16 can use both insect-derived and plant-derived oils with equal efficiency for growth and P(3HB) accumulation. The trend was consistent with previous observations using PO and BSFLO. High P(3HB) yields were achieved using mixtures. Biomass decreased marginally with BSFLO alone. Regardless of the carbon source used, whether CPKO, PO, or BSFLO, the H16 strain synthesized only 3HB homopolymer, with no incorporation of 3HHx detected. This aligns with its wild-type genetic background, which lacks the necessary pathways to produce medium-chain-length monomers.

**Fig. 4:**
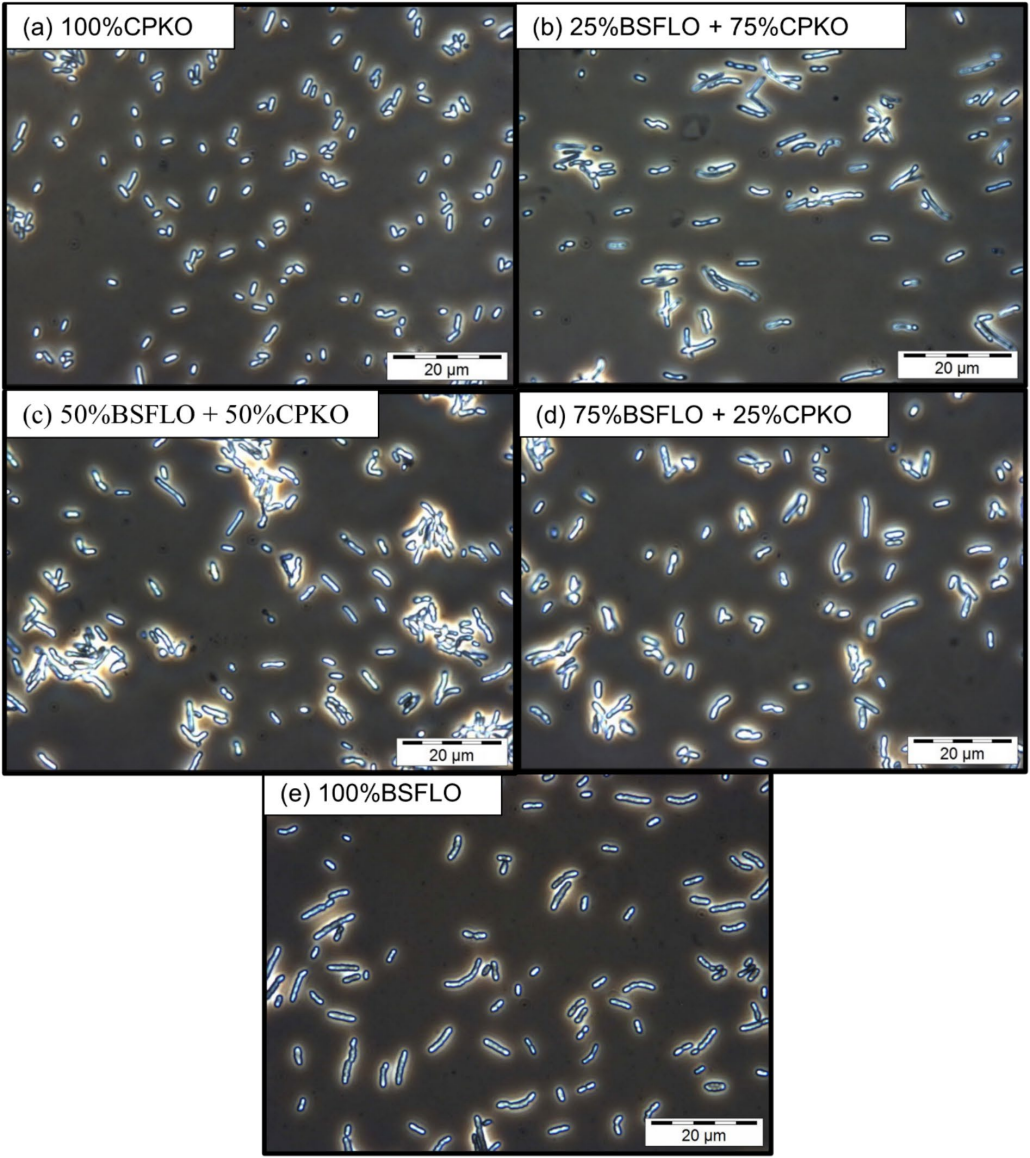
Phase contrast microscope images at 1000 × magnification of P(3HB) in *C. necator* H16 using (**a**) 100% CPKO, (**b**) 25% BSFLO + 75% CPKO, (**c**) 50% of BSFLO + 50% CPKO, (**d**) 75% of BSFLO + 25% CPKO and (**e**) 100% of BSFLO.

Phase contrast microscopy at 1000 × magnification (Fig. 4) supported these observations confirmed the findings, with visible intracellular PHA granules in all samples. The cells grown on 100% CPKO (Fig. 4a) and its mixtures (Fig. 4b-d) appeared denser, whereas cells cultured on 100% BSFLO (Fig. 4e) appeared more regular. Granule brightness and size were comparable across treatments. This indicates PHA accumulation was not affected by carbon source composition, which is per the results shown in Table 5. These results demonstrate the versatility of *C. necator* H16 in producing P(3HB) from various lipid-based substrates, including BSFLO.

#### Biosynthesis of P(3HB-*co*-3HHx) copolymer by *C. necator* Re2058/pCB113 using CPKO and BSFLO as carbon sources

The biosynthesis of P(3HB-*co*-3HHx) by *C. necator* Re2058/pCB113 was studied using CPKO, BSFLO, and their mixtures as sole carbon sources at 10 g/L. As shown in Table 6, all substrates supported both copolymer production and cell growth, with dry cell weight (DCW) ranging from 8.35 ± 0.81 g/L for 100% BSFLO to 9.87 ± 0.30 g/L for the 75% CPKO + 25% BSFLO blend. Statistical analysis showed that the 100% BSFLO treatment showed significantly lower biomass, but PHA content was statistically similar across treatments, ranging from 73.9 ± 1.26 wt.% to 85.7 ± 1.13 wt.%. The recombinant strain exhibited similar biomass trends with both PO and CPKO, achieving the highest DCW when 50% BSFLO was mixed with 50% PO (10.39 g/L) or CPKO (9.87 g/L). No significant differences were observed between the DCW obtained with PO (10.33 g/L) and CPKO (9.21 g/L) when used as sole carbon sources.

**Table 6:** Polyhydroxyalkanoate production in *C. necator* Re2058/pCB113 using different carbon sources^a^.

Carbon Sources (10 g/L)	Dry Cell Weight (g/L)	PHA Content^b^ (wt.%)	PHA Composition^b^ (mol%)	
			3HB	3HHx
CPKO 100%	9.21 ± 0.08^xy^	85.7 ± 1.1^x^	80 ± 1	20 ± 1
CPKO 75% + BSFLO 25%	9.87 ± 0.30^x^	76.0 ± 0.6^x^	80 ± 1	20 ± 1
CPKO 50% + BSFLO 50%	9.66 ± 0.31^xy^	75.6 ± 0.3^x^	83 ± 0	17 ± 0
CPKO 25% + BSFLO 75%	9.51 ± 0.79^xy^	81.3 ± 1.4^x^	82 ± 1	18 ± 1
BSFLO 100%	8.35 ± 0.81^y^	73.9 ± 1.3^x^	82 ± 1	18 ± 1

^a^The cells were cultivated in 50 mL MM supplemented with 10 g/L of oil as carbon source and 0.54 g/L of urea as nitrogen source at 30 °C, 200 rpm for 48 h. The values reported are means ± standard deviations from triplicate cultures. The values indicated by different superscript alphabets (x, y) are significantly different (Tukey’s HSD test, p˂0.05).

^b^Determined by Gas chromatography.

PHA, polyhydroxyalkanoate; 3HB, 3-hydroxybutyrate; 3HHx, 3-hydroxyhexanoate; CPKO, Crude palm kernel oil; BSFLO, Black soldier fly larval oil.

In terms of monomer composition, the 3HHx content ranged from 17 mol% to 20 mol%, with no clear variation in trends among the treatments. This differs from the previous study (Table 3) using PO and BSFLO with the same strain, where even partial replacement of BSFLO (25%) led to a higher 3HHx incorporation. In that case, the 3HHx content increased from 14 mol% in 100% PO to 15–16 mol% in the blends and 18 mol% in 100% BSFLO, and the increase was statistically significant.

The 3HHx monomer content obtained from PO and PO-based substrates (14–16 mol%) was generally lower than that from CPKO and CPKO-based media (17–20 mol%). This difference could be attributed to the distinct fatty acid compositions of the two oils, with CPKO being richer in lauric acid, which is more favourable for PHA biosynthesis. Similar results were observed in the current CPKO and CPKO-based treatments, particularly the 25:75 BSFLO: CPKO blend, which yielded 20 mol% 3HHx.

The 3HHx levels were already high (20 mol%) in 100% CPKO, likely due to its abundance of medium-chain fatty acids, reducing the visible effect of BSFLO addition on monomer enrichment. BSFLO alone produced a PHA content of 73.9 wt.% and 3HHx at 18 mol%, indicating that this recombinant strain effectively utilizes both plant- and insect-derived lipids for copolymer synthesis.

**Fig. 5:**
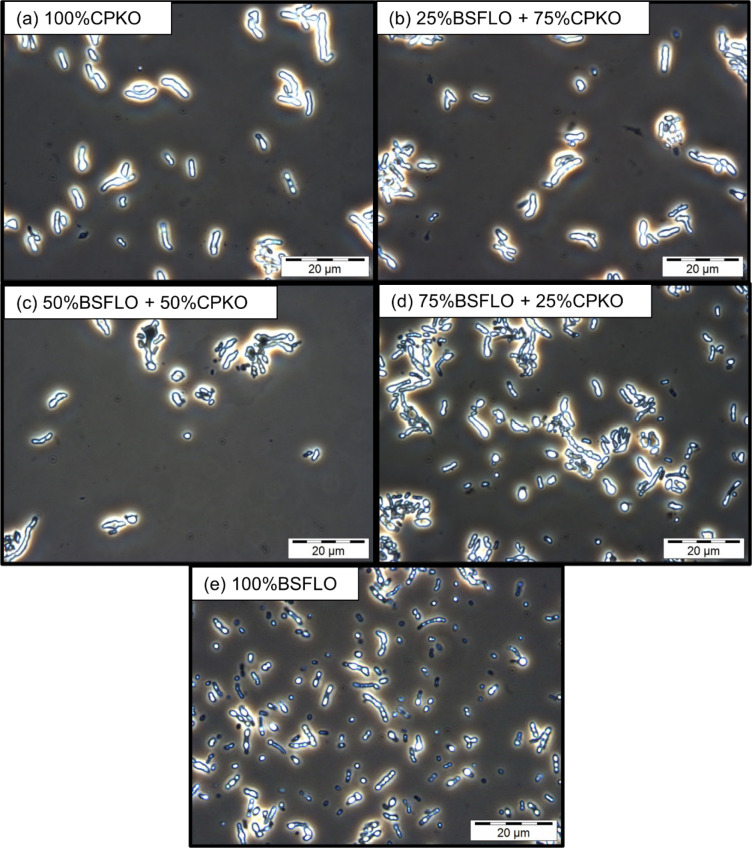
Phase contrast microscope images at 1000 × magnification of P(3HB-*co*-3HHx) in Re2058/pCB113 using (**a**) 100% CPKO, (**b**) 25% BSFLO + 75% CPKO, (**c**) 50% of BSFLO + 50% CPKO, (**d**) 75% of BSFLO + 25% CPKO and (**e**) 100% of BSFLO.

The microscopy images (Fig. 5) confirmed the presence of intracellular PHA granules in all conditions. Cells grown on 100% CPKO (Fig. 5a) and mixtures thereof (Fig. 5b-d) were more clumped and denser, while cells in 100% BSFLO culture (Fig. 5e) were dispersed but maintained bright inclusion bodies that indicated PHA accumulation. No apparent variations in PHA content and DCW were observed between cultures grown on 100% palm-based oils and BSFLO mixtures. This indicates that BSFLO incorporation did not adversely affect growth or polymer production.

#### Biosynthesis of P(3HB-*co*-3HHx) copolymer by *C. necator* Re2160/pCB113 using CPKO and BSFLO as carbon sources

To evaluate its potential for copolymer production, the biosynthesis of P(3HB-*co*-3HHx) using *C. necator* Re2160/pCB113 was investigated with CPKO, BSFLO, and their blends as carbon sources at 10 g/L. Table 7 revealed that all carbon sources supported PHA production, though DCW and PHA content differed significantly. The highest DCW was 6.80 g/L with 100% CPKO; the lowest, 3.24 g/L, was with 100% BSFLO. According to Tukey’s HSD test, biomass yield decreased significantly as the proportion of BSFLO increased. Similarly, PHA content decreased from 73.6 wt.% to ~ 61 wt.% in blends containing higher BSFLO. While cell growth and total PHA yield were reduced with higher BSFLO concentrations, the incorporation of 3HHx monomers increased. The 3HHx content rose from 28 mol% in 100% CPKO to 30–31 mol% in BSFLO-containing mixtures, suggesting that BSFLO contributed effectively to 3HHx formation.

**Table 7:** Polyhydroxyalkanoate production in *C. necator* Re2160/pCB113 using different carbon sources^a^.

Carbon Sources (10 g/L)	Dry Cell Weight (g/L)	PHA Content^b^ (wt.%)	PHA Composition^b^ (mol%)	
			3HB	3HHx
CPKO 100%	6.80 ± 0.70^x^	73.6 ± 0.7^x^	72 ± 1	28 ± 1
CPKO 75% + BSFLO 25%	4.61 ± 0.30^xy^	61.2 ± 0.9^x^	69 ± 1	31 ± 1
CPKO 50% + BSFLO 50%	4.43 ± 0.40^y^	61.3 ± 1.3^x^	70 ± 1	30 ± 1
CPKO 25% + BSFLO 75%	4.01 ± 0.33^y^	62.9 ± 0.2^x^	71 ± 0	29 ± 0
BSFLO 100%	3.24 ± 0.17^y^	64.5 ± 0.6^x^	72 ± 1	28 ± 1

^a^The cells were cultivated in 50 mL MM supplemented with 10 g/L of oil as carbon source and 0.54 g/L of urea as nitrogen source at 30 °C, 200 rpm for 48 h. The values reported are means ± standard deviations from triplicate cultures. The values indicated by different superscript alphabets (x, y) are significantly different (Tukey’s HSD test, p˂0.05).

^b^Determined by Gas chromatography.

PHA, polyhydroxyalkanoate; 3HB, 3-hydroxybutyrate; 3HHx, 3-hydroxyhexanoate; CPKO, Crude palm kernel oil; BSFLO, Black soldier fly larval oil.

**Fig. 6:**
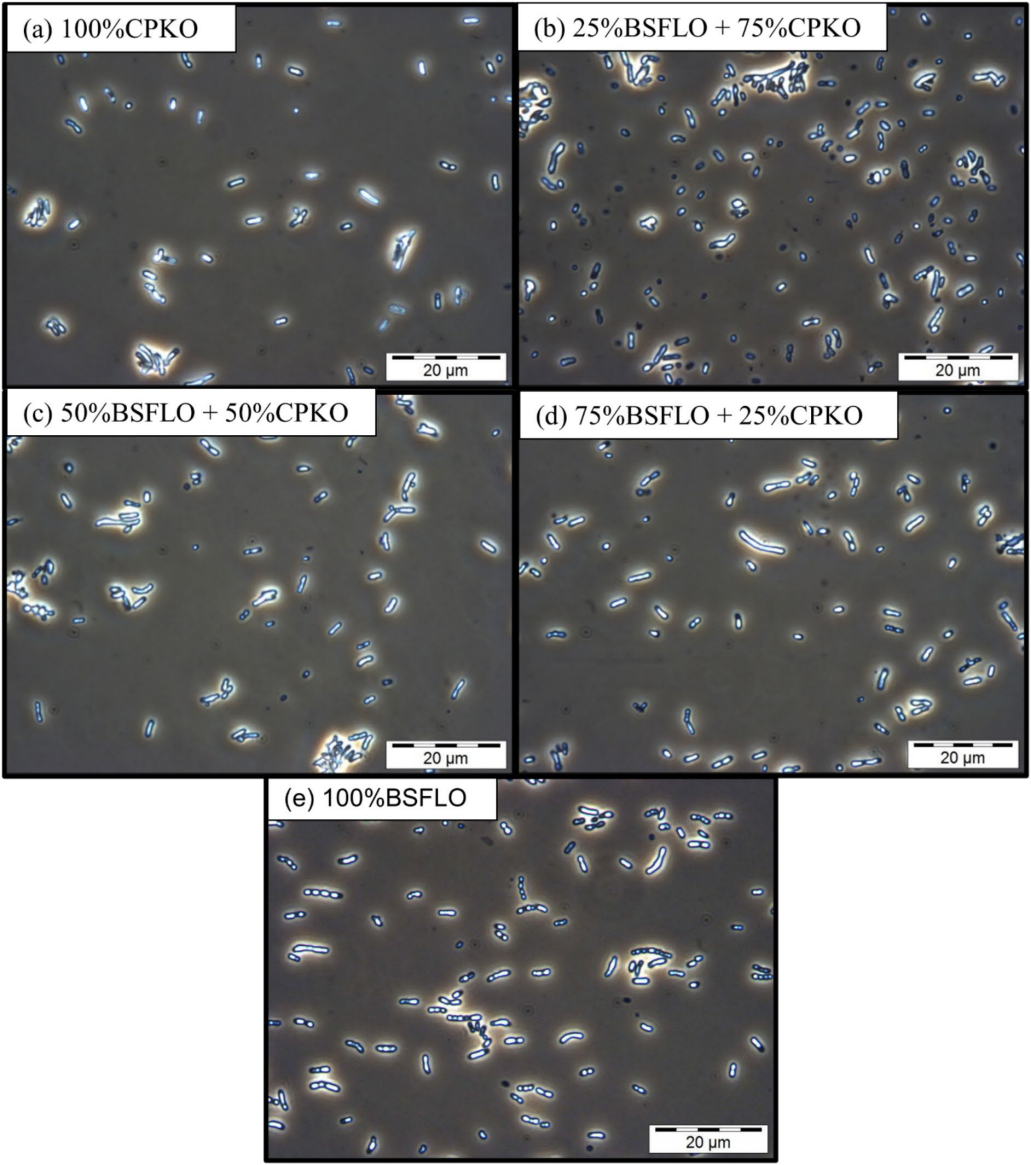
Phase contrast microscope images at 1000 × magnification of P(3HB-*co*-3HHx) in Re2160/pCB113 using (**a**) 100% CPKO, (**b**) 25% BSFLO + 75% CPKO, (**c**) 50% of BSFLO + 50% CPKO, (**d**) 75% of BSFLO + 25% CPKO and (**e**) 100% of BSFLO.

This pattern is similar to previous work with PO-BSFLO blends in the same strain (Table 4), where BSFLO accelerated the 3HHx content but at the expense of lowering DCW. However, CPKO is rich in medium-chain fatty acids, primarily lauric acid, higher than in BSFLO. Therefore, adding BSFLO caused a less dramatic increase in 3HHx formation. Phase contrast microscopy, as shown in Fig. 6, confirmed the presence of intracellular PHA granules under all conditions. Cells grown in all treatments (Fig. 6a–e) showed similar cell morphology and granule appearance, with consistently visible inclusion bodies, which correlates well with the relatively stable PHA content observed across the carbon sources. These findings confirm that although BSFLO may reduce biomass and total PHA yield, but it enhances 3HHx incorporation. Visual accumulation of PHA was not impaired, supporting BSFLO as a valuable substrate.

*Re2160/pCB113 exhibited* a distinct trend compared to the other strains. Although its DCW and PHA content were significantly lower than those of the other two strains for every substrate, it produced the highest 3HHx composition, particularly with BSFLO and its mixtures (24–31 mol%). In both recombinant strains, the C6 intermediate is converted into hydroxyhexanoyl-CoA by the enzyme PhaJ, forming (*R*)-3-hydroxyhexanoyl-CoA (3HHx-CoA). The presence of a broad-substrate-specificity PhaC polymerase allows this monomer to be incorporated into PHA copolymers^25^.

In Re2160/pCB113, however, the disrupted phaCAB operon leads to an accumulation of C4 intermediates and a reduced availability of 3HB-CoA, favouring the synthesis and incorporation of 3HHx monomers. The deletion of the phaB gene limits the conversion of acetoacetyl-CoA to 3HB-CoA, reducing 3HB supply^25^. Additionally, excess free CoA in the cytoplasm inhibits PhaA activity, further slowing 3HB-CoA synthesis. Together, these metabolic changes explain why Re2160/pCB113 produces PHA with a higher 3HHx fraction despite lower overall biomass and polymer content.

As a result, Re2160 not only incorporates a higher proportion of 3HHx but also exhibits a reduced growth rate, limited carbon utilization, and lower molecular weight of the resulting polymer^25,27^. This explains why Re2160/pCB113 outperformed Re2058/pCB113 in 3HHx incorporation but showed overall lower biomass and productivity. For instance, using 25% BSFLO with 75% CPKO yielded 31 mol% 3HHx, higher than PO-based substrates (22–26 mol%). This suggests that BSFLO and CPKO provide more medium-chain monomers due to higher lauric acid content.

It is also worth noting that for *C. necator* Re2160/pCB113, the PHA content remained relatively stable across all types of carbon sources and their mixtures, including PO, BSFLO, PO + BSFLO, and CPKO + BSFLO. The values consistently fell within the range of 58.9–71.0 wt.% for PO-based combinations, and 61.2–73.6 wt.% for CPKO-based and BSFLO alone. These minor fluctuations were not statistically significant, suggesting that BSFLO and its mixtures can produce PHA content comparable to that obtained with palm oil-based substrates (PO and CPKO). Although the DCW for Re2160/pCB113 was comparatively lower when cultivated on BSFLO and its mixtures (ranging from 3.24 to 4.61 g/L), BSFLO still demonstrated strong potential for 3HHx enrichment.

Overall, Re2160/pCB113 maintains moderate polymer productivity while enhancing 3HHx incorporation on BSFLO and CPKO substrates.

The utilization of different lipid-based carbon sources, such as CPKO, PO, BSFLO, and their mixtures, showed distinct trends in DCW, PHA content, and monomer composition across strains. The highest DCW, 10.74 g/L, aligns with theoretical yields (~ 1.38 g-P(3HB)/g-carbon), confirming efficient PHA synthesis from 10 g/L oil^56^.

A previous study^57^ reported that *C. necator* H16 grown on 10 g/L of animal fats and tallow achieved DCW values of 4.0–4.5 g/L and PHA contents of 72–75% (w/w), though biomass and polymer yields were much lower when using mixed animal fats or cattle tallow (DCW 2.5–3.1 g/L, PHA 56–61%).

In contrast, the present study shows that BSFLO and its mixtures significantly outperformed these animal fats. When fed 10 g/L of BSFLO or its blends, *C. necator* H16 reached DCW values of 8.52–10.59 g/L, with PHA content between 79.0% and 86.2%. These results demonstrate that BSFLO not only supports higher biomass but also yields substantially more PHA compared to traditional animal fats.

Similarly, the Re2058/pCB113 strain previously^58^ showed limited growth on some animal fats, particularly poultry fat, with DCW ranging from 1.5 to 4.6 g/L and PHA content of 48–71.9% (w/w). In this study, using BSFLO and its mixtures, the same strain achieved DCW of 8.35–10.39 g/L and PHA content of 69.2–84.3%. While earlier studies^58^ reported higher 3HHx content (16–27%), BSFLO still enabled effective incorporation of 3HHx (15–20%) along with high PHA yields, highlighting its strong potential as a feedstock for copolymer production.

Also, according to the research^59^ the *Salinivibrio* sp. M318 strain was able to accumulate a dry cell weight (DCW) of 5.8 g/L when waste fish oil was used as the sole carbon source. When a mixture of glycerol and waste fish oil was used, the strain achieved 7.8 g/L DCW, with P(3HB) content reaching 42 wt.%. This is much lower in both DCW and PHA accumulation compared to the current study, where BSFLO and its mixtures resulted in higher yields. These results suggest that BSFLO supports better yields than a mixture of glycerol and waste fish oil or waste fish oil alone, making BSFLO a more efficient carbon source for PHA production.

The findings of this study demonstrate that the BSFLO has great potential as a feedstock in the biopolymer industry, particularly in the production of both homopolymer and copolymer with diverse monomer compositions. BSFL’s significance lies not just in its sustainability profile but also in its biochemical nature, given that BSFLO is rich in lauric acid (C12), which via β-oxidation generates C6 intermediates, precursors for 3HHx. The copolymers are crucial since they integrate *scl-* and *mcl-*PHA, providing a unique combination of properties. The *scl-mcl* PHAs offer enhanced flexibility and strength, suitable for diverse industrial applications^60^. This adaptability enables the development of materials that are both strong and flexible, which is a significant benefit in many industries.

### Characterization of PHA from BSFLO

PHA characterization from *C. necator* H16, Re2058/pCB113, and Re2160/pCB113 cultivated on 100% BSFLO revealed distinct variations in monomer composition, molecular weight, and thermal properties (Table 8). As would be expected, the wild-type strain H16 synthesized a homopolymer of P(3HB) with no detectable 3HHx incorporation, whereas Re2058/pCB113 and Re2160/pCB113 incorporated 3HHx at 18 mol% and 28 mol%, respectively, confirming the functional integration of the metabolic pathway for medium-chain monomer biosynthesis in these recombinant strains.

**Table 8:** The molecular weight and thermal properties of PHA obtained from BSFLO as the sole carbon source.

Bacterial Strains	3HHx Monomer Composition	Molecular Weights (× 10^5^ Da)			Thermal Properties (°C)	
		*M*_n_^a^	*M*_w_^b^	*M*_w_/*M*_n_^c^	*T*_g_^d^	*T*_m_^e^
H16	0	3.5 ± 0.2	15.1 ± 0.1	4.3 ± 0.2	1.0	171
Re2058/pCB113	18	1.1 ± 0	3.5 ± 0	3.3 ± 0	−4.4	127
Re2160/pCB113	28	0.8 ± 0	1.6 ± 0	1.9 ± 0	−3.2	81, 106

^a^Number-average molecular weight.

^b^Weight-average molecular weight.

^c^Polydispersity index.

^d^Glass transition temperature.

^e^Melting temperature.

3HHx, 3-hydroxyhexanoate.

The incorporation of 3HHx affected the molecular weight of the polymer. H16 produced the highest molecular weights, with a number-average molecular weight (*M*_n_) of 3.5 × 10^5^ Da and a weight-average molecular weight (*M*_w_) of 15.1 × 10^5^ Da, resulting in a polydispersity index (PDI, *M*_w_/*M*_n_) of 4.3. Copolymers from Re2058/pCB113 and Re2160/pCB113 exhibited lower molecular weights. Re2058/pCB113 showed *M*_n_ of 1.1 × 10^5^ Da and *M*_w_ of 3.5 × 10^5^ Da, with a PDI of 3.3, while Re2160/pCB113 recorded the lowest values, *M*_n_ of 0.8 × 10^5^ Da and *M*_w_ of 1.6 × 10^5^ Da, with a narrower PDI of 1.9. The decrease in molecular weight with higher 3HHx incorporation is consistent with previous studies, attributed to reduced crystallinity and irregular chain packing^61^.

The DSC analysis shows the range of *T*_m_ obtained was between 81 °C and 171 °C. Typically, with a high monomer fraction value, the *T*_m_ decreased from 171 °C (0 mol% 3HHx) to 81 °C (28 mol% 3HHx), confirming that increasing 3HHx enhances amorphous character. The double melting peaks in the latter can be explained by differences in side-chain length and by recrystallization^9,62^.

Padermshoke et al. (2004)^63^ reported similar dual melting transitions in P(3HB-*co*-15 mol% 3HHx), suggesting that the dual peaks correspond to phase transitions between crystalline and amorphous regions. According to North & Jenkins (2025)^64^, this thermal behavior is further attributed to the formation of secondary lamellae, where the first peak reflects the melting of the original crystalline domains and the second peak corresponds to the melting of newly formed lamellae. In the present study, the glass transition temperature (*T*_g_) shifted from 1.0 °C in the P(3HB) homopolymer to − 4.4 °C at 18 mol% 3HHx, indicating increased chain mobility due to the incorporation of bulky 3HHx monomers^65^. Notably, at 28 mol% 3HHx, the *T*_g_ modestly increased to − 3.2 °C, likely due to intermolecular interactions or increased cross-linking^66^, which suggests partial restriction of chain mobility and phase reorganization within the polymer matrix.

Overall, 3HHx incorporation decreases molecular weight, crystallinity, and thermal stability, confirming its role in tailoring the flexibility and mechanical performance of P(3HB-*co*-3HHx) copolymers for diverse bioplastic applications.

## Conclusion

The potential of BSFLO as a sole carbon source for PHA production was evaluated using *C. necator* H16 and two recombinant strains (Re2058/pCB113 and Re2160/pCB113). The results showed that BSFLO successfully supported PHA biosynthesis, achieving a 3-hydroxyhexanoate (3HHx) molar fraction of up to 28%, demonstrating its ability to enhance copolymer flexibility and tailor mechanical performance. Saturated fatty acids such as lauric acid (C12:0) and palmitic acid (C16:0) play a crucial role in enhancing PHA production by serving as precursors for monomer formation through the β-oxidation pathway, thereby promoting cellular polymer accumulation. The high lauric acid content of BSFLO promotes 3HHx formation, indicating that saturated medium-chain fatty acids enhance PHA yield and composition control. This study was based on BSFLO obtained from a single source and extraction method, which may limit generalizability. Therefore, future studies should investigate the influence of BSFLO origin and extraction processes on composition and PHA productivity to confirm and broaden these findings.

## Data Availability

Data is provided within the manuscript or supplementary information files.

## Abbreviations

BSFL: Black soldier fly larvae
BSFLO: Black soldier fly larvae’s oil
PO: Palm olein
CPKO: Crude palm kernel oil
PHA: Polyhydroxyalkanoate
P(3HB): Poly(3-hydroxybutyrate)
P(3HB-*co*-3HHX): Poly(3-hydroxybutyrate-*co*-3-hydroxyhexanoate)
DCW: Dry cell weight
FAME: Fatty acid methyl ester
MM: Mineral medium
GC: Gas chromatography
GPC: Gel permeation calorimetry
CME: Caprylic methyl ester
DSC: Differential scanning calorimeter
*M*_w_: Weight-average molecular weight
*M*_n_: Number-average molecular weight
*M*_w_/*M*_n_: Polydispersity index
*T*_g_: Glass transition temperature
*T*_m_: Melting temperature
